# Microsynteny is a powerful front for microbial strain tracking

**DOI:** 10.1016/j.crmeth.2024.100862

**Published:** 2024-09-16

**Authors:** Peiwen Cai, Tal Korem

**Affiliations:** 1 Program for Mathematical Genomics, Department of Systems Biology, Columbia University Irving Medical Center, New York, NY, USA; 2 Department of Obstetrics and Gynecology, Columbia University Irving Medical Center, New York, NY, USA

## Abstract

Genomic diversity within species can be driven by both point mutations and larger structural variations, but so far, strain-tracking approaches have focused mostly on the former. In a recent issue of *Nature Biotechnology*, Ley and colleagues^1^ introduce SynTracker, a tool designed for scalable strain tracking with microsynteny in low-coverage metagenomic settings.

## Teaser

Genomic diversity within species can be driven by both point mutations and larger structural variations, but so far, strain-tracking approaches have focused mostly on the former. In a recent issue of *Nature Biotechnology*, Ley and colleagues introduce SynTracker, a tool designed for scalable strain tracking with microsynteny in low-coverage metagenomic settings.

## Main text

Understanding complex microbiomes and microbial interactions with their environments or hosts often requires high-resolution profiling of the diverse microbes involved. Over the years, researchers have reported strains of the same species with different functional or phenotypic associations across clinical contexts. For example, certain strains of *Staphylococcus aureus* with antibiotic resistance and enterotoxin genes were found to be associated with poor healing in diabetic foot ulcers.^2^ There is, therefore, a growing appreciation of the need for reliable and robust methods for microbial strain inference. In the big data era of metagenomics, in which numerous strains are potentially detectable across many samples, a scalable tool is especially desirable.

Resolving strain phylogenies has been notoriously challenging due to high genomic similarity within species, manifesting as sparse signals in genomic variation that distinguish conspecific strains. Variations in genomic sequences often come from point mutations or structural variations, underlying different types of genomic events. Point mutations are typically captured as single-nucleotide variations (SNVs), while structural variations are measured on the kilobase pair (kbp) scale and include a broad spectrum of events, such as insertion/deletion (indel), recombination, and horizontal gene transfer, all of which can shuffle the order of genetic sequences during evolution. While SNVs have been used for strain inference,^3^^,^^4^^,^^5^ they require higher sequencing coverage and need to address sequencing errors. Detection of changes in sequence order, termed “synteny analysis,” can also be used for phylogenetic inference, in a manner complementing SNV detection in strain inference, with the potential to reveal different evolutionary patterns. Synteny can be further divided by scale: macrosynteny investigates long-range order conservation among a large number of genetic loci, and microsynteny focuses on a smaller local set. In practice, however, both SNV and synteny analyses are challenging to apply at scale due to the many all-against-all sequence comparisons required. To reduce computing time and resources, several methods have focused on select reference marker genes, which may not be available or well curated for all species.

In a recent work published in *Nature Biotechnology*,^1^ Enav, Paz, and Ley presented a solution to these challenges: SynTracker, a bioinformatic tool for microbial strain tracking. SynTracker incorporates microsynteny-based analysis into strain phylogenetic inference and tracking in a scalable manner suitable to metagenomic sequencing data. Furthermore, SynTracker can provide robust inference with only a fraction of the genome and is independent of gene annotations, making it appealing for low-coverage taxa in metagenomic data.

**Figure 1:**
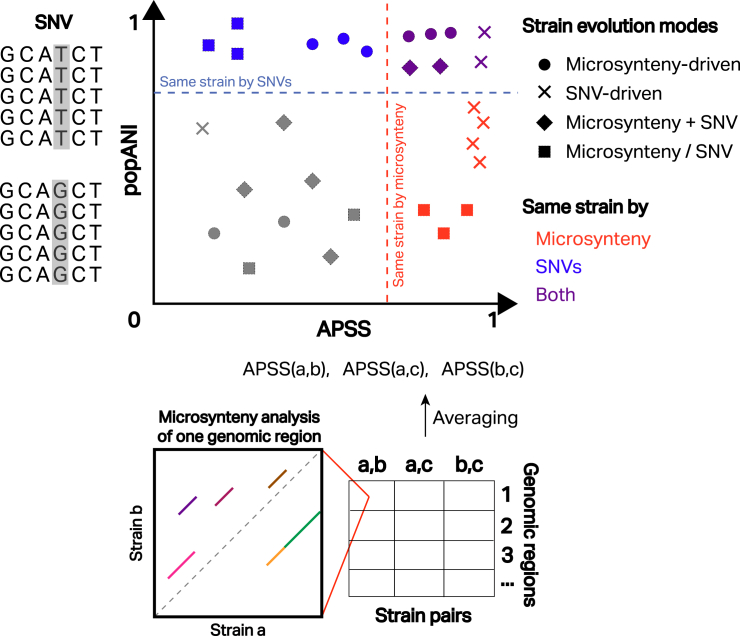
Combining microsynteny with SNVs reveals possible strain-evolution patterns An illustration of an analysis combining SNV- and microsynteny-based metrics for strain inference. A population-level SNV is detected between two samples by inStrain^3^ (top left). The population average nucleotide identity (popANI) metric captures the conservation of such SNVs between pairs of strains. SynTracker calculates APSS values among three pairs of strains as the average of synteny scores across a number of 5-kbp-long genomic regions (bottom). Synteny blocks for one genomic region between a strain-pair alignment are illustrated on the left, whereas colored diagonal lines demonstrate homologous regions in different locations. Plotting both popANI and APSS per strain pair on a scatterplot (top middle) illustrates how the two measurements can reveal strain similarities and different evolutionary patterns.

In order to detect synteny, SynTracker starts by fragmenting a reference genome into 5-kbp regions. It then performs homology search via local alignment between the center 1 kbp of each such region and a set of metagenomic sequencing samples. These regions serve as anchors, facilitating retrieval of the 4 kbps around the homologs identified, which are then aligned between strain pairs from different samples (Figure 1). Not only does this step improve scalability by aligning fragments instead of whole genomes, it is also independent of gene annotations.

For each aligned pair of bins, blocks of continuously aligned sequences, or synteny blocks, are detected (Figure 1). A genomic region that is located in a different position would not be continuously aligned to its surrounding regions, creating a new synteny block. Thus, the number of synteny blocks reflects the number of rearrangement events. A synteny score is calculated such that it is higher the fewer synteny blocks are present and lower if less sequence is covered by synteny blocks, indicating lower overall similarity. A final sample-pair-level metric, the average pairwise synteny score (APSS), is calculated as the average of all scores across a number of homologous regions. The authors demonstrated that APSS can serve as a similarity metric that reflects phylogeny and, interestingly, showed that robust inference can be done using only a fraction of the genome. Specifically, APSS values from approximately 20% of the *Escherichia coli* genome, or about 200 randomly selected 5-kbp regions, generated a phylogenetic tree in concordance with a previously published one,^6^ and similar results persist even when coverage is decreased to as low as ∼2% of the genome. In another analysis, pairwise APSS values were shown to be consistent with the phylogeny of five *Nitrosococcus oceani* strains derived from a previously published gene-based synteny analysis,^7^ this time with regions spanning 28% of the genome.

By construction, APSS detects the difference in sequence block orders (microsynteny) and is thus insensitive to SNVs. Enav, Paz, and Ley demonstrated this key property using genomes simulated to have evolved exclusively under either single-nucleotide polymorphisms (SNPs) or indel events. In this analysis, the APSS values remained close to 1 across the SNP-only simulations while declining expectedly in the indel-only set, even though overall sequence identity was lower in the SNP-only simulations. This analysis provides an important intuition for the envisioned use of this tool: combining the microsynteny-based signals from SynTracker with SNV-based ones from other tools would enable better strain inference and tracking, potentially also providing insight into different evolution patterns.

The authors demonstrated this approach through a diverse set of analyses. They ran SynTracker alongside inStrain,^3^ a strain-detection tool based on SNVs, on four species and revealed different possible evolutionary patterns: point mutations only, structural variations only, or a mix of both (Figure 1). While some species (*Neisseria gonorrhoeae*) might undergo both point mutations and structural variations simultaneously, others (*Streptomyces rimosus*) can have subpopulations within a single host that mostly rely on just one strategy. These analyses, as well as additional analyses in a gut microbiome dataset collected across three countries, revealed a spectrum of evolutionary strategies in different strains, from hypermutators (point mutation heavy) to hyper-recombinators (structural variation heavy), and made a compelling case for considering both point mutations and structural variants in characterizing genomic diversity within species. Using the APSS values, the authors also reported that while some species exhibited geographically specific strain similarities, others were shared across the three countries.

Finally, the authors tested the effectiveness of SynTracker as a strain-tracking tool, comparing it with three existing metagenomic strain-tracking tools, all SNV based: MIDAS,^4^ StrainPhlAn,^5^ and inStrain.^3^ While both MIDAS and StrainPhlAN rely on reference marker genes for SNV detections, inStrain and SynTracker can use information from the entire reference genomes for SNV and microsynteny analyses, respectively. The authors performed a benchmark of strain classification in gut metagenomic samples of premature infant twins in which strains from within a twin pair are considered identical and those between twin pairs are considered different. SynTracker had similar or better results compared with all three tools, using only 40 regions in the APSS calculations, and demonstrated a stronger improvement for tracking phages and plasmids.

This promising demonstration of the potential of microsynteny-based inference in metagenomic data analysis opens the door to exciting future research directions. While the fragmentation and alignment strategies used by SynTracker help reduce the computational burden of microsynteny detection, recent developments in rapid large-scale sequence alignment, such as MMSeqs2,^8^ can potentially improve its scalability while allowing for a more comprehensive genome-wide synteny analysis along with reduced coverage requirements for metagenomic analysis. With improved scalability, it would be interesting to see whether incorporating larger-scale variations,^9^ or macrosynteny, can further improve SynTracker’s detection power; similarly, approaches that operate without reliance on reference or linear genomes^10^ may be helpful. Additionally, investigating optimal thresholds for strain delineation using microsynteny across scenarios and ecosystems, as well as systematic integration with SNV-based or even macrosynteny-based approaches, is likely to facilitate even better phylogenetic and strain-tracking analysis. As the application of synteny to microbiome data is still at an early stage, it could be worth exploring other tools and approaches, such as SynChro^11^ and MCScanX,^12^ although adaptation or optimization may be required from eukaryotes to prokaryotes. Interestingly, SynChro computes reciprocal best hits for a more stringent homology search, potentially impacting synteny detection in prokaryotes, which exhibit more divergent sequence shuffling. Finally, a deep understanding of biases affecting synteny-based analyses, including library depth, mappability, and pangenome openness or size, would assist future analyses.

Overall, SynTracker is a robust and scalable microsynteny-based strain-tracking tool for metagenomic data. Moving beyond reference marker genes and requiring only a fraction of the genome, it has the flexibility to work with microbial elements that have relatively lower sequencing coverage, such as phages and plasmids. This can help researchers tap into less-studied microbial domains. Combining SynTracker with an SNV-based strain-inference or strain-tracking tool at a similar scope can even allow discovery of diverse strain patterns, providing researchers with a closer look at the evolutionary dynamics in microbial communities. This exciting front in strain inference and tracking is likely to fuel new discoveries in microbiome research.

## Declaration of interests

The authors declare no competing interests.
